# Advanced glycation end (AGE) product modification of laminin downregulates Kir4.1 in retinal Müller cells

**DOI:** 10.1371/journal.pone.0193280

**Published:** 2018-02-23

**Authors:** Kayla Thompson, Jonathan Chen, Qianyi Luo, Yucheng Xiao, Theodore R. Cummins, Ashay D. Bhatwadekar

**Affiliations:** 1 Department of Ophthalmology, Eugene and Marilyn Glick Eye Institute, Indiana University School of Medicine, Indianapolis, Indiana, United States of America; 2 Department of Biology, Indiana University-Purdue University, Indianapolis, Indiana, United States of America; University of Florida, UNITED STATES

## Abstract

Diabetic retinopathy (DR) is a major cause of adult blindness. Retinal Müller cells maintain water homeostasis and potassium concentration via inwardly rectifying Kir4.1 channels. Accumulation of advanced glycation end products (AGEs) is a major pathologic event in DR. While diabetes leads to a decrease in the Kir4.1 channels, it remains unknown whether AGEs-linked to the basement membrane (BM) affect normal Kir4.1 channels. For this study, we hypothesized that AGE-modification of laminin is detrimental to Kir4.1 channels, therefore, disrupting Müller cell function. The AGE-modified laminin-coated substrates were prepared by incubating Petri-dishes with laminin and methylglyoxal for seven days. The rat Müller cells (rMC-1) were propagated on AGE-modified laminin, and Kir4.1 expression and function were evaluated. Quantification of AGEs using ELISA revealed a dose-dependent increase in methylglyoxal-hydro-imidazolone adducts. The rMC-1 propagated on AGE-modified laminin demonstrated a decrease in Kir4.1 levels in immunofluorescence and western blot studies and a decrease in the Kir4.1 channel function. Kir4.1 decrease on AGE-modified laminin resulted in a disorganization of an actin cytoskeleton and disruption of α-dystroglycan-syntrophin-dystrophin complexes. Our studies suggest that AGE-modification of laminin is detrimental to Kir4.1 channels. By studying the role of AGEs in Kir4.1 channels we have identified a novel mechanism of Müller cell dysfunction and its subsequent involvement in DR.

## Introduction

Diabetic retinopathy (DR) is a leading cause of vision impairment and blindness in adults that affects roughly seven million individuals in the United States [1]. The combination of high metabolic demand and nominal vascular supply limits the retina’s ability to assimilate to the metabolic stress of diabetes. The retina is the most vulnerable tissue affected by diabetic milieu due to its intricate network of vascular and neural cells that require high oxygen demand with limited vascularity [2]. Diabetes immensely enhances the accumulation of advanced glycation end products (AGEs) through the glycation of proteins thereby disrupting molecular conformation, altering enzymatic activity, and interfering with receptor function. AGEs create cross-links with proteins, lipids and nucleic acids accumulating first extracellularly through the basement membrane (BM), and then intracellularly leading to the development of diabetic complications [3, 4]. The accumulation and increased formation of AGEs in retinal neurons, the vasculature, and the BM of the retina play a critical role in the development of DR [5–7]. BM-modification by AGEs is detrimental to integrin signaling resulting in dysfunction of the retinal neurovascular unit leading to diabetic complications such as DR [8].

Müller cells are an integral component of the retinal cellular environment [9]. Müller glia span across the entire thickness of the retina to maintain the stability of the retinal extracellular environment by regulating potassium levels, uptaking neurotransmitters, storing glycogen, creating an electrical insulation of receptors and other neurons, and acting as mechanical support between retinal neurons, retinal blood vessels, and the vitreous humor [10]. Müller cells regulate K^+^ balance via inwardly rectifying Kir4.1 channels. Notably, the polarized pattern of Kir4.1 exhibits a strong decrease in perivascular regions in diabetic retinas expressing Müller cell markers [11, 12]. Our studies demonstrate that Müller cells exhibit a functional clock with a diurnal rhythm of Kir4.1 and diabetes disturbs the natural circadian rhythm of Kir4.1 [13]. AGEs are known to induce Müller cell dysfunction [14], but it remains unknown how AGE-modification of BM affects Kir4.1 channels in diabetes.

In the present study, we examine the effects of laminin, a critical BM component that contributes to cell attachment, differentiation, migration, and adhesion to promote cell survival. Previous studies suggest that laminin is required for normal Kir4.1 expression in Müller cells [15]. However, it is unknown whether AGE-modification of laminin is detrimental to Kir4.1 expression. We hypothesized that AGE-modification of the laminin leads to a decrease in Kir4.1 channels, thereby resulting in Müller cell dysfunction. Methylglyoxal (MGO) is a dicarbonyl compound generated by cell metabolism, glucose oxidation and lipid peroxidation [16, 17]. This highly reactive compound undergoes a rapid modification in hyperglycemic conditions such as diabetes leading to the formation of irreversible AGEs [17, 18]. We used MGO as an AGE-intermediate to prepare AGEs *in vitro*. The laminin-coated Petri-dishes were incubated with MGO for seven days and then quantified for AGEs. Additionally, we tested Kir4.1 expression and function in rat Müller cells (rMC-1) propagated on AGE-modified laminin. Our studies demonstrate that AGE-modification of laminin downregulates Kir4.1 expression due to disorganization of the actin cytoskeleton and disruption of α-dystroglycan-syntrophin-dystrophin complexes.

## Methods

### Culture of rMC-1

The rat Müller cells (rMC-1; #RRID: CVCL_8140) were obtained as a gift from Dr. Vijay Sarthy, Northwestern University and Dr. Timothy S. Kern, Case Western Reserve University, Cleveland, OH. The rMC-1 cells were cultured in T-75 flasks in Dulbecco Modified Eagle Medium (DMEM) supplemented with 10% fetal bovine serum (FBS) and antibiotic-antimycotic (Gibco-Life Technologies, Thermo Fishers, Grand Island, NY). The rMC-1 cells were authenticated using rat short tandem repeat (STR) analysis, interspecies contamination test and mycoplasma at IDEXX Bioresearch, Columbia, MO. The rMC-1 cells were found to be negative for mycoplasma and the samples were confirmed to be of rat origin without any mammalian interspecies contamination. In addition, the rMC-1 cells demonstrated a prominent band of glutamine synthase-1 (GS-1) in western blot studies (S1 Fig)

### Preparation of AGE-modified laminin

AGE-modified laminin was prepared by coating Petri-dishes with laminin (20 μg/ml; Sigma-Aldrich, St. Lois, MO) for 2 hours at 37°C followed by incubating Methylglyoxal (MGO) (10, 100 & 1000 μM; Sigma-Aldrich, St. Lois, MO) for 7 days at 37°C. The AGE substrates were washed with PBS to remove any excess methylglyoxal and unbound adducts.

### Quantification of AGE adducts

AGE-modified laminin was prepared in microcentrifuge tubes similar to AGE-modified substrates. The glycated and control proteins were dialyzed against 30 mmol/l ammonium formate, pH 7.8 and 4°C. AGEs were quantified via detection of methylglyoxal-hydro-imidazolone (MG-H1) adducts using Methylglyoxal Competitive ELISA. (Cell Biolabs, Inc., San Diego, CA)

### Western blotting

The rMC-1 cells were pelleted and lysed in RIPA buffer (Sigma-Aldrich, St. Louis, MO). Protein Concentration was estimated using BCA assay (Pierce, Thermo Scientific, Rockford, IL), and equal amounts of proteins (30 μg) were loaded and separated on 4–12% Bis-Tris gels (Novex, Life Technologies, Carlsbad, CA). Proteins were transferred to a PVDF membrane (Life Technologies), which was blocked with 5% milk or BSA as per the antibody requirement. The following antibodies were used for probing: Kir4.1 goat-rabbit (1:2000; Alomone Labs, Jerusalem, Israel), glutamine synthase -1, (GS-1; 1:500, EMD-Millipore, Burlington, MA), TWIK (1:200; Alomone labs), α-Tubulin goat-mouse (1:5000; Sigma-Aldrich, St. Louis, MO) and β-actin (1:5000: Sigma-Aldrich) antibodies. The PVDF membranes were incubated with the aforementioned primary antibodies overnight at +4°C. The following set of antibodies were used as a secondary loading control: HRP goat-mouse for α-Tubulin (1:100; Sigma-Aldrich), and HRP goat-rabbit for Kir4.1 (1:2000; Sigma-Aldrich). The secondary antibody incubations were performed for two hours at room temperature. The bands were visualized using ECL2 western blotting substrate (Pierce, Thermo Scientific, Rockford, IL) on an XRS gel documentation system with Quantity One software (Bio-Rad, CA).

### Immunofluorescence of Kir4.1

rMC-1 cells were fixed in 4% paraformaldehyde for 20 minutes followed by 5 minutes of D-PBS in 0.05% Tween solution. The cells were blocked for 2 hours in 2% Goat Serum followed by incubation with primary antibody Kir4.1 (1:200; Alomone Labs) overnight at ^+^4°C. The primary antibody was removed by three total washes with D-PBS in 0.05% Tween. The secondary antibody staining was performed using fluorescent-conjugated Alexa fluor 488 (1:500; Invitrogen-Molecular Probes, ThermoFischer, Rockford, IL) incubated for two hours at room temperature. Finally, the slides were mounted in a Slow fade DAPI mounting media and imaged via a confocal microscope (Zeiss LSM 510 META; Carl Zeiss MicroImaging GmbH, Jena, Germany) and analyzed through Zen11 software (Zeiss, Thornwood, NY).

### Electrophysiological recordings

The constructs pcDNA3.1-Kir4.1 (9.5 μg) and pMax-EGFP (0.5 μg) were transiently co-transfected into rMC-1 cells using the Lipofectamine 2000 (Invitrogen-Life Technologies). Cells were seeded on glass coverslips on control or AGE-matrix and incubated at 37°C for 24 hrs prior to patch-clamp recording.

Whole-cell voltage-clamp recordings were performed at room temperature (~21°C) using an EPC-10 amplifier and the Pulse program (HEKA Electronics). Fire-polished electrodes (1.0–2.0 MΩ) were fabricated from 1.7 mm capillary glass using a P-97 puller (Sutter Instruments). The pipette solution contained (in mM): 140 KCl, 1.1 EGTA, 10 NaCl and 10 HEPES, pH 7.3. The bathing solution was (in mM): 140 NaCl, 3 KCl, 1 mm MgCl_2_, 1 CaCl_2_, and 10 HEPES, pH 7.3 (adjusted with NaOH). The offset potential was zeroed before contacting the cell. After establishing the whole-cell recording configuration, the resting potential was held at -60 mV for 5 min to allow adequate equilibration between the micropipette solution and the cell interior. Membrane currents were usually filtered at 5 kHz and sampled at 20 kHz. Voltage errors were minimized using 70%-80% series resistance compensation.

### Staining for actin cytoskeleton in rMC-1 cells

rMC-1 cells were propagated on 8-well chamber slide coated with AGE-modified laminin for 24 hours and fixed using 4% paraformaldehyde for 20 minutes. Filamentous actin (F-actin) were stained using Phalloidin antibodies (Invitrogen-Molecular Probes, ThermoFischer, Rockford, IL) to visualize the f-actin. The confocal images of f-actin were imaged via a confocal microscope (Zeiss LSM 510 META; Carl Zeiss MicroImaging GmbH, Jena, Germany) and analyzed through Zen11 software (Zeiss, Thornwood, NY).

### Quantification of α-Dystroglycan complexes in rMC-1

rMC-1 cells were propagated on 8-well chamber slide coated with AGE-modified laminin for 24 hours. The primary antibody was directly added to media and incubated for thirty minutes at +4°C using anti-α-Dystroglycan (1:100; EMD Millipore, Darmstadt, Germany) followed by secondary staining using fluorescently conjugated antibodies Alexa fluor 488 (1:400) for one hour at room temperature. The slide was washed in PBS and mounted in SlowFade gold with DAPI mounting media and imaged using a confocal microscope (Zeiss LSM 510 META; Carl Zeiss MicroImaging GmbH, Jena, Germany) and analyzed through Zen11 software (Zeiss, Thornwood, NY). Quantification of α-Dystroglycan complexes were performed by counting hyperfluorescent spots using image-analysis software (ImageJ; National Institutes of Health, Bethesda, MD; available by ftp @ zippy.nimh.nih.gov/ or at http://rsb.info.nih.gov/nih-image; developed by Wayne Rasband, National Institutes of Health, Bethesda, MD).

### Syntrophin and dystrophin colocalization analysis

rMC-1 cells were propagated on 8-well chamber slides coated with AGE-modified laminin overnight at 37°C. All treatment groups were stained simultaneously. The cells were blocked for 2 hours in 2% Goat Serum and then incubated with both anti-syntrophin (1:200, Abcam, Cambridge, MA) and anti-dystrophin (1:200 ab15277, Abcam) primary antibodies overnight at +4°C. The primary antibodies were removed by three total washes with D-PBS in 5% Tween. The secondary stain was added by incubating fluorescent-conjugated antibodies Alexa 488 (1:500) and Alexa 594 (1:500) for syntrophin and dystrophin, respectively, for two hours at room temperature. The cells were mounted in a Slow fade DAPI mounting media and imaged via a confocal microscope (Zeiss LSM 510 META) and analyzed through Zen11 software (Zeiss, Thornwood, NY). The confocal images were captured by keeping confocal settings constant across the treatment groups. Determination of the colocalization coefficients was performed using the image-analysis software, Icy (Institut Pasteur, Paris, France). First, the Thresholder plugin was used to automatically create an intensity threshold for each image. Then, the colocalization coefficients were calculated using the colocalization studio plugin.

### Statistics

All data were expressed as mean ± SEM. Statistical analysis was performed using a Graph Pad-Prizm 6 (GraphPad Software, La Jolla, CA) software. The normality of data was tested using either a D'Agostino & Pearson normality test or Shapiro-Wilk normality test prior to undertaking parametric testing of statistical analysis. The statistical significance was evaluated using Student’s t-test or a One-Way ANOVA followed by a Tukey’s post- hoc test, unless and otherwise specified. Each experiment was repeated at least n = 3 times unless and otherwise specified. The data were considered statistically significant when the p-value was less than 0.05.

## Results

### AGEs show a concentration-dependent increase following methylglyoxal treatment

**Fig 1:**
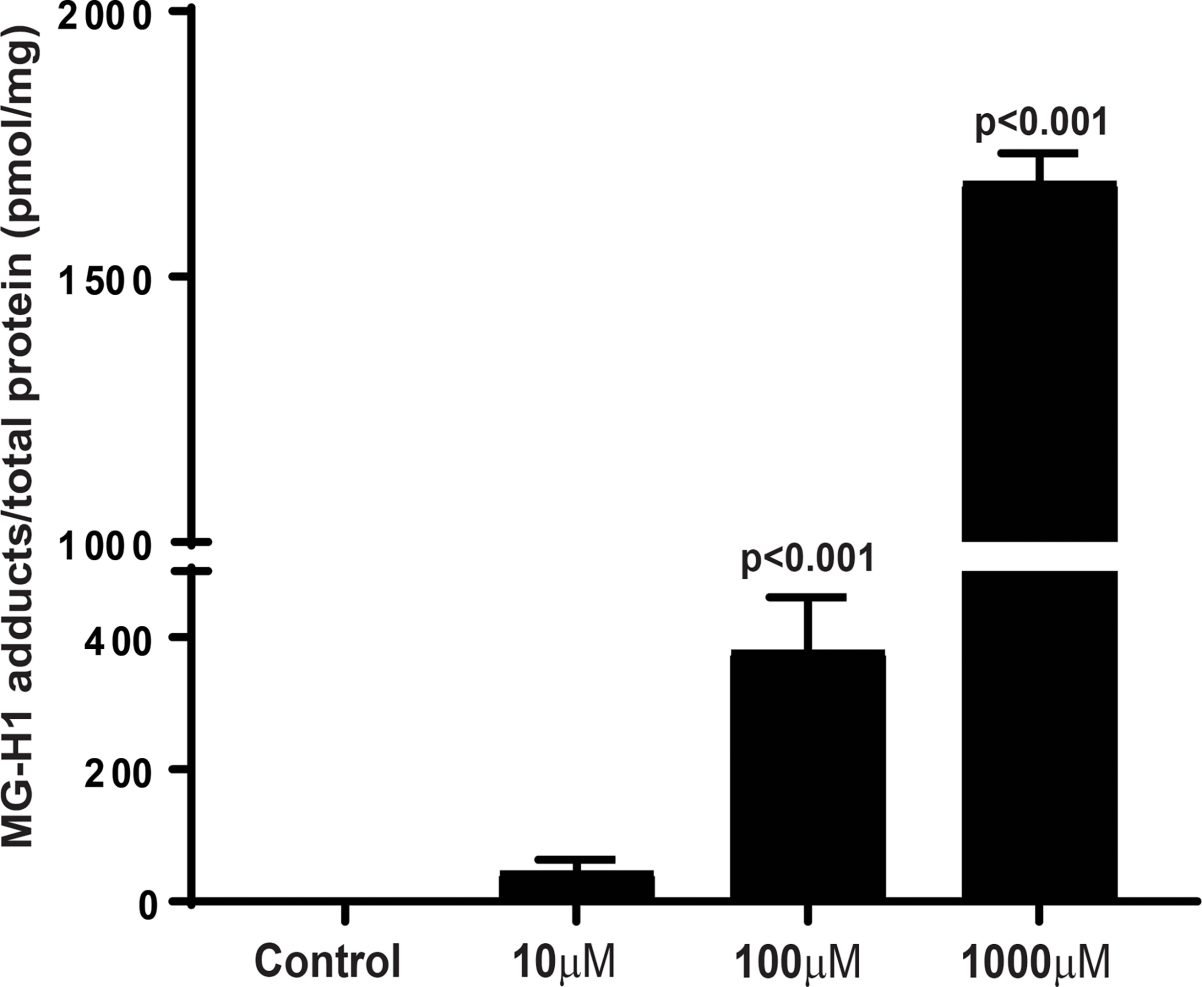
Quantification of AGEs using ELISA. Laminin was incubated with methylglyoxal and AGEs were quantified as methylglyoxal-hydro-imidazolone (MG-H1) adducts using competitive ELISA. Bar chart showing a concentration-dependent increase in MG-H1 adducts after treatment with methylglyoxal, n = 7.

AGEs were quantified as methylglyoxal-hydro-imidazolone (MG-H1) adducts using competitive ELISA. MG-H1 is the AGE formed from the methylglyoxal intermediate. We observed a concentration-dependent increase in MG-H1 adducts after treatment with methylglyoxal (10–1000 μM). The linear regression analysis exhibited a concentration-dependent increase in MG-H1 adducts (p<0.05). The 100 μM (380.2 ± 30.3) and 1000 μM (1680 ± 23.6) groups differed significantly as compared to control group, however, for 10 μM (46.1 ± 6.5), this difference was statistically insignificant (Fig 1).

### AGE-modification of laminin leads to a decrease in Kir4.1 protein expression

**Fig 2:**
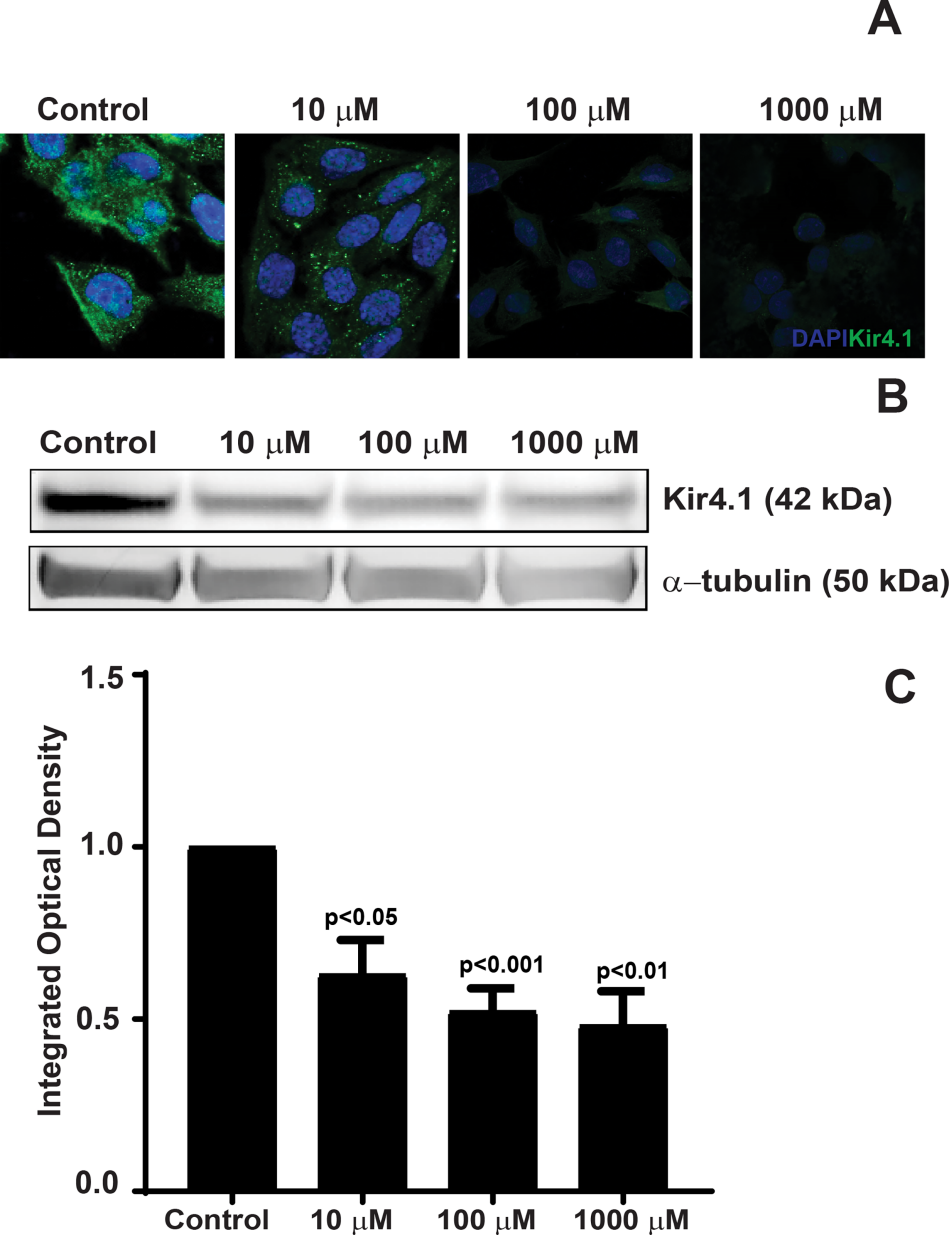
Decrease in Kir4.1 protein expression in rMC-1 cells propagated on AGE-modified laminin. rMC-1 cells were plated on AGE-modified laminin and the Kir4.1 expression was determined using immunofluorescence and western blot. (**A**) rMC-1 cells were stained with Kir4.1 antibodies followed by secondary staining with Alexa 488. The immunofluorescence staining of Kir4.1 revealed a profound decrease in the Kir4.1 expression on AGE-modified substrates as compared to untreated laminin. (**B**) Representative western blot showing a decrease in Kir4.1 expression. (**C**) Bar chart showing a quantification of the western blots, n = 4.

To study the effect of AGE-modified laminin on Kir4.1 expression, the rMC-1 cells were propagated on varying concentrations of AGE-modified laminin. The AGE-modification of laminin did not affect the viability of rMC-1 cells (S2 Fig). Western Blot and immunofluorescence studies were performed to determine Kir4.1 protein expression. Immunofluorescence imaging of rMC-1 cells stained Kir4.1 antibodies revealed a significant decrease in the Kir4.1 expression on AGE-modified substrates as compared to untreated laminin (Fig 2A); thereby suggesting that AGE-modification of the BM affects Kir4.1 expression. Further, the western blot demonstrated a negative correlation between Kir4.1 protein expression and the concentration of AGE-modified laminin (Fig 2B). In order to quantify the western blot data, the integrated optical density (IOD) was determined for respective Kir4.1 and α-tubulin bands. The IOD of treatment groups was determined by normalizing the data to an untreated group. We observed a 1.6-fold decrease (0.63 ± 0.09; p<0.05) in Kir4.1 expression in cells propagated on 10 μM AGE-modified laminin. A 1.9-fold decrease (0.52 ± 0.06; p<0.01) was observed in cells propagated on 100 μM and for a 1000 μM group this decrease was about 2-fold (0.48 ± 0.09 p<0.01).

### AGE-modification of laminin causes a decrease in Kir4.1 function

**Fig 3:**
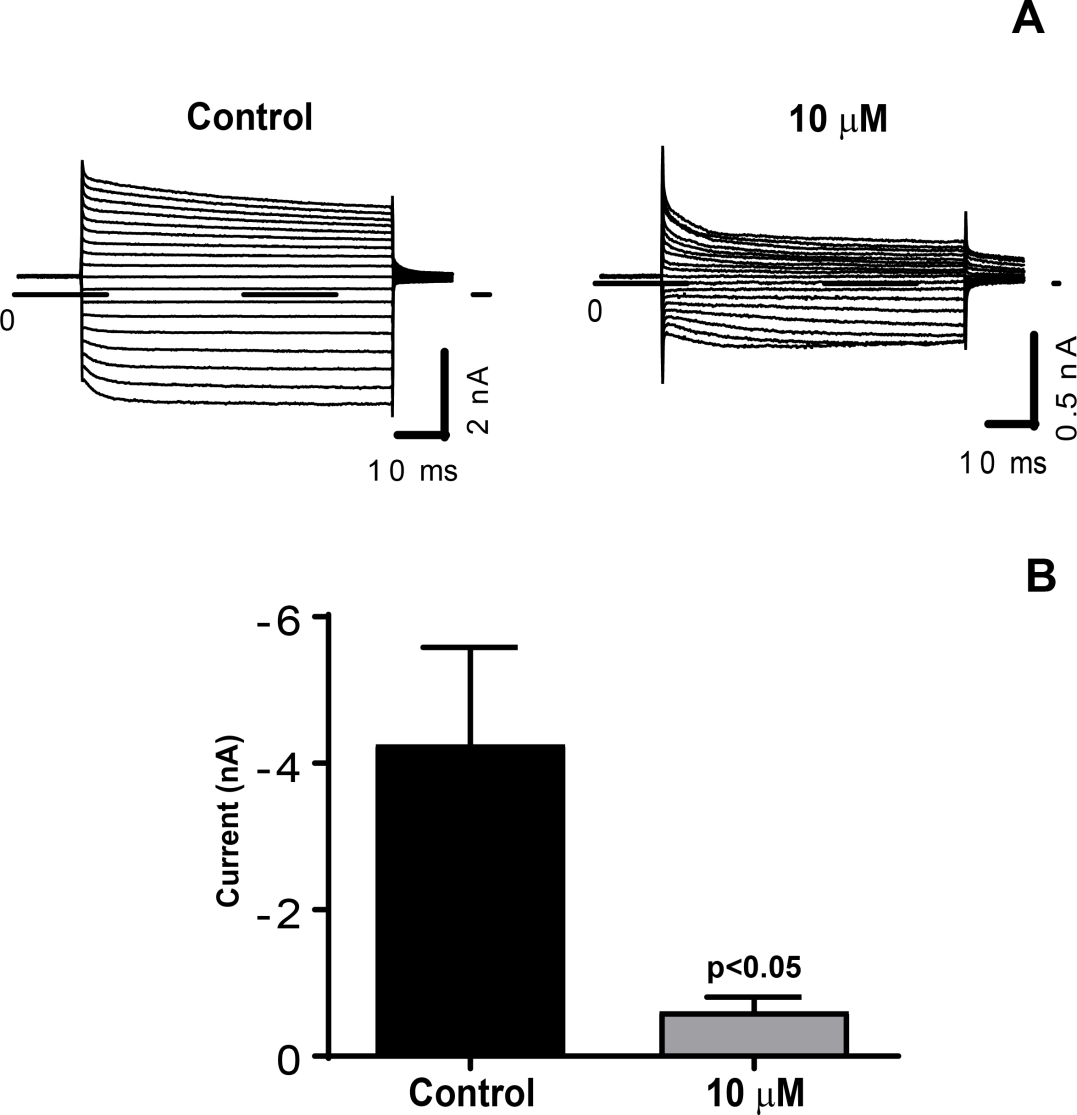
AGE-modification of laminin leads to a decrease in Kir4.1 function. (**A**) Representative current traces of Kir4.1 currents under control and AGE-modified matrix (10 μM). Currents were elicited by applying 50-ms depolarizing potentials ranging from -140 mV to +30 mV from a holding potential of -60 mV (10-mV increment). Dash line indicates zero-current level. (**B**) Mean amplitude of the inward potassium currents elicited at -140 mV (control, n = 7; 10 μM, n = 9). p < 0.05.

Next, we tested whether the decrease in Kir4.1 expression reflects a functional change in the K^+^ channel, we performed the whole-cell recordings for Kir4.1 currents. As the rMC-1 cells did not evoke Kir4.1 currents by itself, we first transfected rMC-1 cells transiently with pcDNA3.1-Kir4.1 and pMax-EGFP. The whole cell Kir4.1 currents were determined on rMC-1 cells seeded on control and 10 μM AGE-modified laminin. We observed a decrease in Kir4.1 currents on AGE-modified laminin (Fig 3A) and there was a significant decrease (contol = -4.25 ± 1.33; 10 μM = -0.60 ± 0.19; p<0.05) in mean amplitude on AGE-modified laminin (Fig 3B).

### AGE-modification of laminin results in disorganization actin cytoskeleton and a decrease of α-Dystroglycan complexes

**Fig 4:**
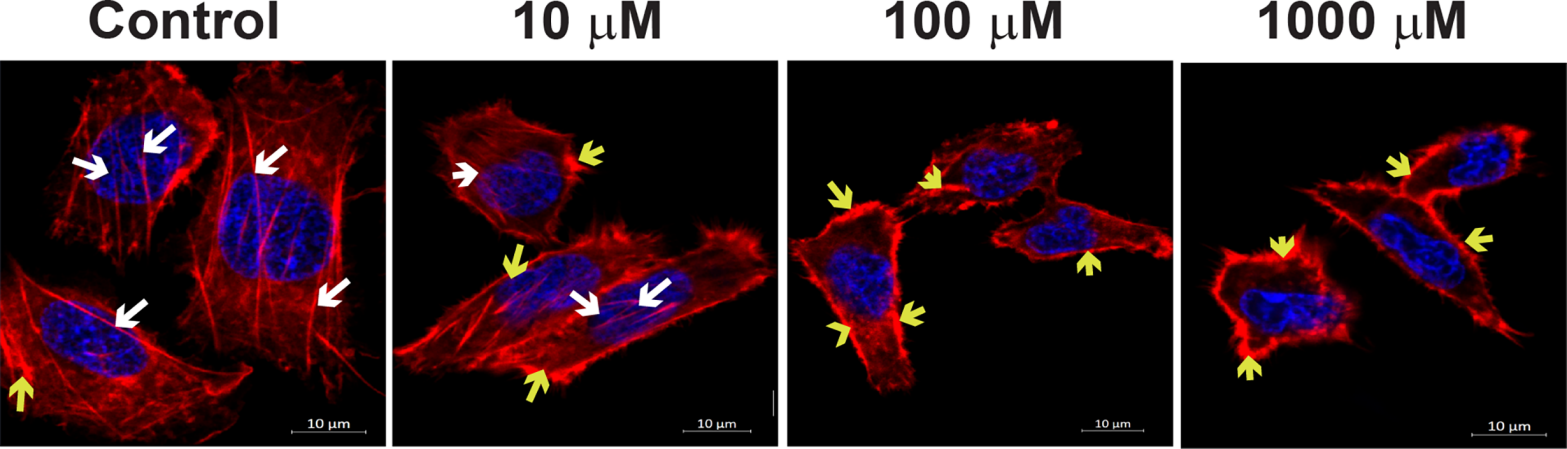
AGE-modification of laminin results in disorganization actin cytoskeleton in rMC-1 cells. rMC-1 cells were propagated on AGE-modified laminin and stained for the phalloidin antibodies. The untreated cells exhibited continuous trans cytoplasmic actin filaments (white arrow). The treatment of rMC-1 cells on AGE-modified laminin resulted in disorganization of actin skeleton and the actin was mostly observed in clumps in the cytoplasm (yellow arrow), n = 3.

We used immunofluorescent imaging to determine how modification of the laminin affects the distribution of the actin cytoskeleton and α-Dystroglycan complexes in correspondence with the decrease in Kir4.1 expression with an increase in treatment. To determine disorganization of the actin cytoskeleton, rMC-1 cells were propagated on AGE-modified laminin and stained with phalloidin antibodies. The treatment of rMC-1 cells on AGE-modified laminin resulted in disorganization of actin skeleton and the actin was mostly observed in clumps in the cytoplasm of rMC-1 cells (Fig 4). These results suggest that BM modification is detrimental to the integrity of the cell.

**Fig 5:**
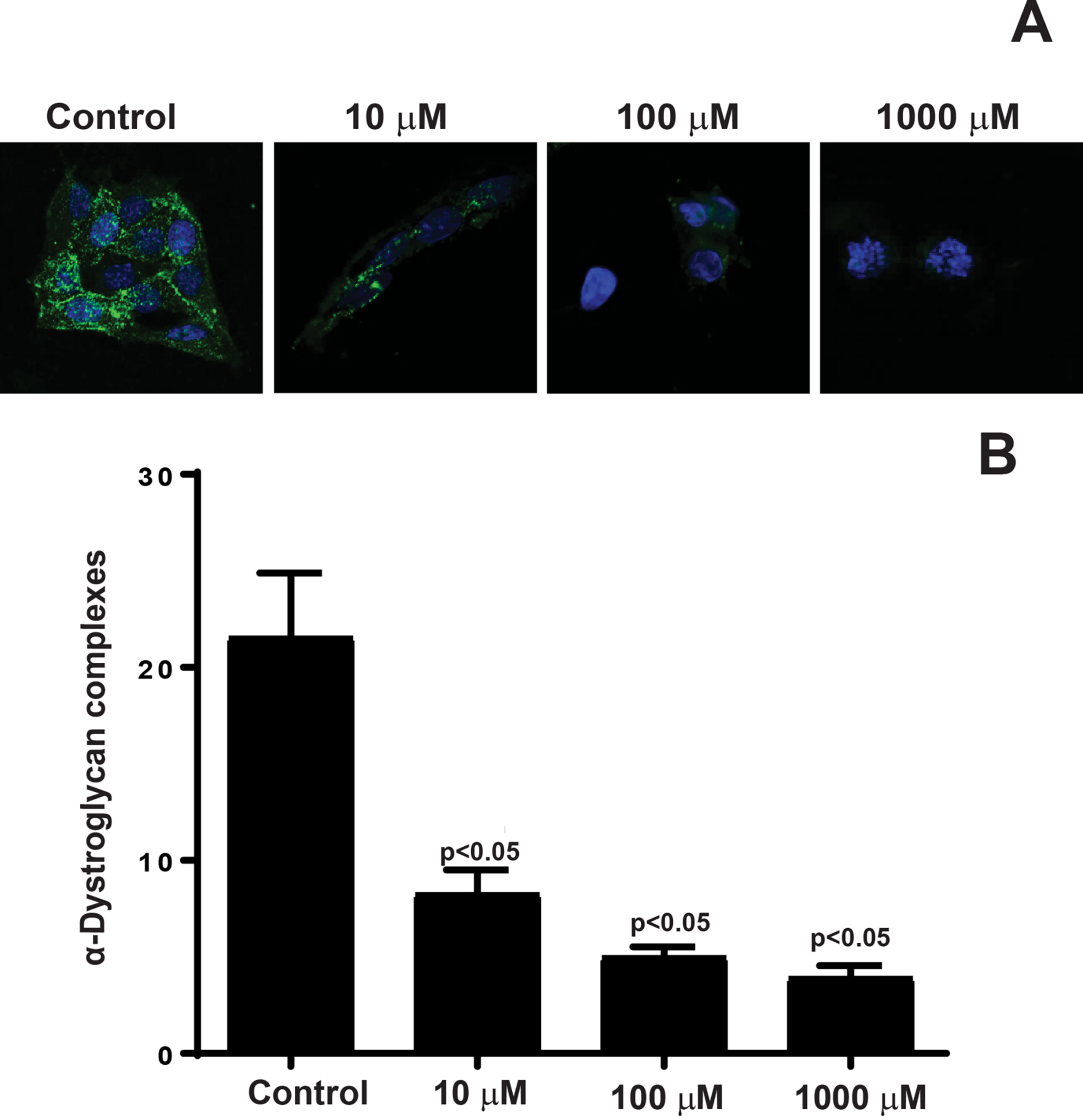
Decrease in α-Dystroglycan complexes in rMC-1 cells propagated on AGE-modified laminin. rMC-1 cells were stained with α-dystroglycan antibodies followed by secondary staining using fluorescently conjugated antibodies. (**A**) Representative photomicrographs showing a decrease in α-dystroglycan expression in rMC-1 cells treated with AGE-modified laminin. (**B**) Quantification of α-dystroglycan complexes revealed a decrease in dystroglycan formation in rMC-1 cells propagated on AGE-modified laminin, n = 3.

Quantification of α-Dystroglycan complexes were determined through immunofluorescent imaging as previously described [19] to provide further information regarding the possible biological mechanism of decrease in Kir4.1 due to AGE-modified laminin. As shown in Fig 5A,α–Dystroglycan complexes decreased significantly with increased AGE-modification. Quantification of the α-Dystroglycan complexes (Fig 5B) revealed a 5.5-fold decrease in complexes between untreated cells (21.5 ± 3.3) and cells plated on AGE-modified laminin 1000μM (3.9 ± 0.6; p<0.05), a 4.3-fold decrease in complexes between untreated and cells plated on AGE-modified laminin 100 μM (5 ± 0.5; p<0.05), and a 2.6-fold decrease in complexes between untreated and cells plated on AGE-modified laminin 10 μM (8.2 ± 1.2; p<0.05). The significant decrease in α-Dystroglycan complexes suggest a strong link between the distribution of these complexes and the expression of Kir4.1.

### AGE-modification of laminin decreases co-localization of syntrophin and dystrophin on AGE-modified laminin

**Fig 6:**
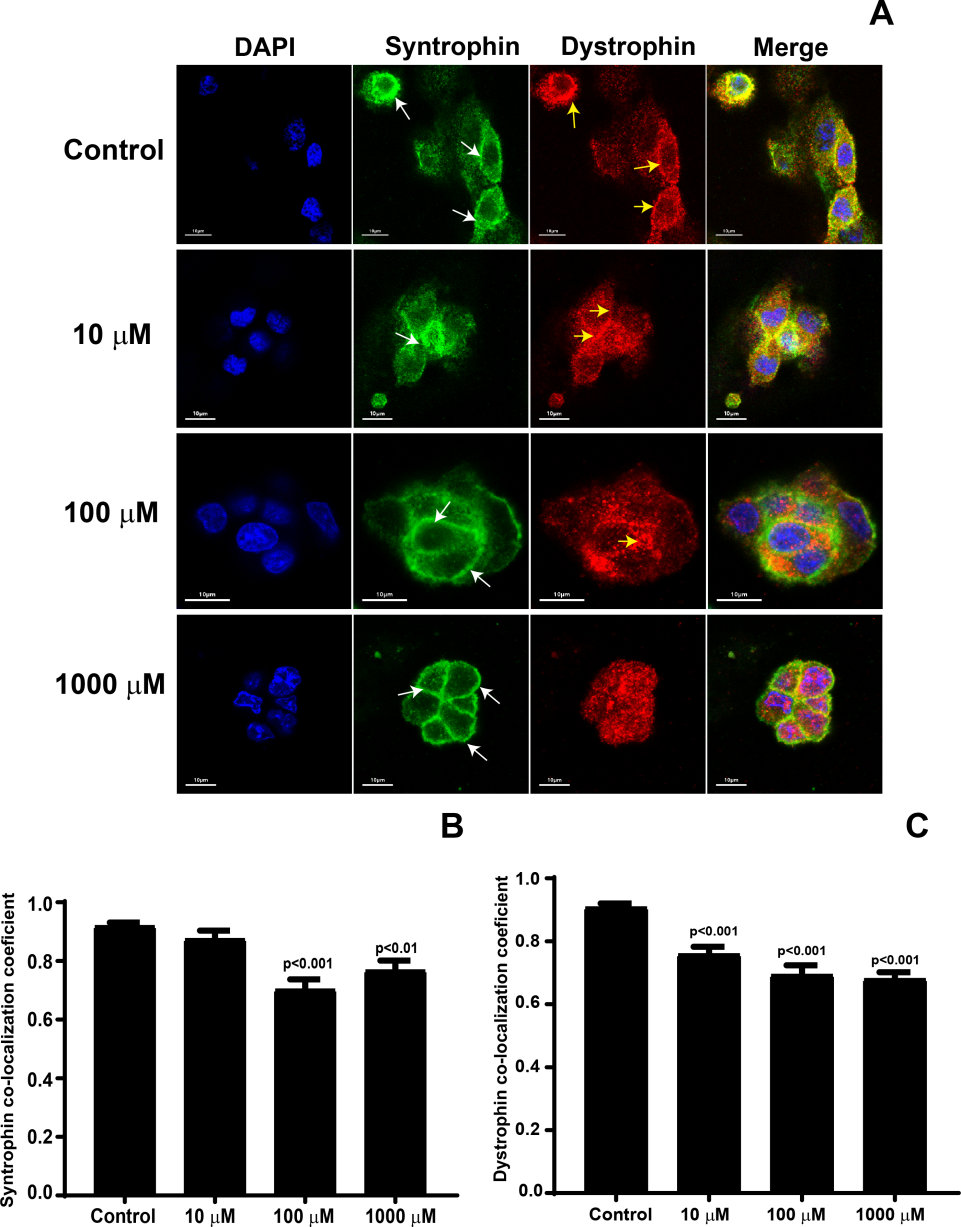
Decrease in colocalization of syntrophin and dystrophin on AGE-modified laminin. rMC-1 cells were propagated on AGE-modified laminin followed by staining with syntrophin and dystrophin antibodies, the images were taken using confocal microscopy. (**A**) Representative photomicrographs showing a decrease in the staining of dystrophin and clustering of syntrophin near cell membrane on AGE-modified laminin. Bar chart showing a Mander’s colocalization coefficient for (**B**) Syntrophin and (**C**) Dystrophin, n = 3.

Previous studies demonstrate that Müller cell binding to laminin anchors α-Dystroglycan complexes followed by redistribution of protein complexes such as syntrophin and dystrophin [19]. After observing a decrease in α-Dystroglycan, we hypothesized that AGE-modification of laminin will further lead to a decrease/distribution of downstream syntrophin-dystrophin protein expression. To test this hypothesis, we co-stained the rMC-1 cells propagated on AGE-modified laminin with syntrophin and dystrophin antibodies. We observed that with an increase in AGE concentration the cytoplasmic distribution of syntrophin clustered towards cell membrane. There was an overall decrease in the dystrophin levels on AGE-modified laminin (Fig 6A). We further quantified the co-localization of syntrophin and dystrophin using Mander’s colocalization coefficient, our studies revealed that there was a significant decrease in syntrophin coefficient at 100 μM (0.70 ± 0.03; p<0.001), and 1000 μM (0.76 ± 0.03; p<0.01) concentration (Fig 6B), however, this decrease was insignificant for 10 μM of AGE-modified laminin (0.87 ± 0.02) when compared to a control group (0.91± 0.01). The dystrophin coefficient was decreased significantly at all the concentrations of methylglyoxal (10 μM = 0.75 ± 0.02; 100 μM = 0.69 ±0.03; 1000 μM = 0.68 ± 0.02; p<0.001; Fig 6C) when compared to a control group (0.90 ± 0.01).

## Discussion

Müller cells play an important role in the retina by maintaining the architecture and high physiological demand. In diabetes, the Müller cells are dysfunctional due to an increased synthesis of glutamate, increase in glial fibrillary acidic protein, and Müller cell swelling due to downregulation of the Kir4.1 channels. Müller cells regulate K^+^ balance via inwardly rectifying Kir4.1 channels. Notably, the polarized pattern of Kir4.1 exhibits a strong reduction in perivascular regions in diabetic retinas [11, 12]. A variety of mechanisms are associated with decreased Kir4.1 expression in diabetes, such an involvement of arachidonic acid pathway [11], increase in VEGF, IL-1β, and ionized calcium binding protein. In addition, AGE accumulation in vascular and neural cells of the retina is a major pathological event in the development of DR [6]. The present study identified a critical role of matrix immobilized AGEs on Müller cell function by demonstrating that AGE-modified laminin affects negatively to Kir4.1 function. Further, we found that the defect in Kir4.1 function was attributed to disruption and disorganization of an actin-dystroglycan-syntrophin-dystrophin complex. Together, the present study demonstrates a novel mechanism of Kir4.1 decease in diabetes by ascertaining the role of basement membrane signaling.

Matrix immobilized AGEs are shown to be detrimental to the retina. Previous studies suggest that bovine retinal pericytes on AGE-modified fibronectin undergo apoptosis and exhibit an increased level of reactive oxygen species [20]. We previously reported that AGE-modification of fibronectin affects the reparative ability of endothelial precursor cells due to dysfunctional integrin-mediated signaling [21]. When the AGE-modified fibronectin was replenished with key peptides known to help with cellular attachment, we observed a reversal of basic cellular functions such as cell attachment, spreading and migration [21]. A similar effect of AGE-modified fibronectin was observed on retinal capillary endothelium and their reparative potential [22]. Laminin is critical to Kir4.1 signal in the Müller cells, Ishi *et al*. previously reported that replenishment of Müller cells with laminin helped to restore Kir4.1 expression which otherwise was lost in tissue culture [15]. Our study further builds on a critical role of basement membrane-mediated signaling by identifying an important role of laminin on Kir4.1 function. Also, our studies further suggest that AGE-modification of laminin results in downregulation TWIK channels (S3 Fig) and speculate that AGE modification of basement membrane may be detrimental to other potassium channels in the retina.

MGO with arginine forms a major adduct, methylglyoxal-derived hydroimidazolone (MG-H), which is known to be increased in lens [23] and retina [24]. We observed a significant increase in AGE adduct (MG-H1) on laminin with increased concentrations of the methylglyoxal. For this study, we used methylglyoxal concentrations in the range of 10–1000 μM which resulted in the formation of MG-H1 adducts in the range of (45–1700 pmol/mg of protein). The MG-H1 adducts measured in our study are line with the previous studies which report MG-H1 levels in the human lens as 4609 pmol/mg of protein [23].

One of the limitations of our study is the use of immortalized cell line, rMC-1 cells in our studies. These cell lines are known to express markers similar to primary culture Müller cells such as CRALBP, GS-1 [25] and have been used in several studies over past years [26–29]. It is noteworthy, that AGE inhibition *in vivo* using pyridoxamine was protective against diabetes-induced Kir4.1 decrease [14]. We believe that our study further enabled understanding of such protective mechanism using robust cell system, such as rMC-1 cells.

Diabetic conditions have been shown to alter K^+^ channels in retinal glial cells causing a decrease of the K^+^ conductance in Müller cells [11]. The decrease in Kir4.1 channels, normally releasing excess K^+^ in the process of spatial K^+^ buffering, causes a disturbance of the retinal K^+^ homeostasis as seen in diabetes-induced dysfunction. We found that intracellular K^+^ absorbance of AGE-modified rMC-1 cells not only proves detrimental to the distribution of Kir4.1, but it also causes a decrease in Kir4.1 function. Our electrophysiological studies support the assertion that there is a decrease in Kir4.1 function on AGE-modified laminin, however, prior Kir4.1-cDNA transfection was necessary to elicit Kir4.1 currents. While acknowledging this limitation we provide an indirect evidence of a decrease in Kir4.1 function and implicate the possibility of an overall decrease in K+ buffering on AGE-modified laminin.

α-dystroglycan is expressed by the Müller cell endfeet where it functions as a laminin receptor and helps in mediating Müller cell interactions with that of the extracellular matrix [19]. Extracellularly, laminin connects to α-dystroglycan which connect to β-dystroglycan to connect intracellularly to dystrophin which binds to the actin cytoskeleton and syntrophin. We found that AGE-modification of laminin resulted in disorganization of the actin cytoskeleton in rMC-1 cells. Furthermore, immunofluorescent imaging demonstrated a profound decrease in α—Dystroglycan complexes and decrease in colocalizaiton of syntrophin-dystrophin complex as AGE-modification of laminin increased, emphasizing critical role of laminin in Kir4.1 expression in Müller cells.

In conclusion, our study has identified a novel mechanism of Müller cell dysfunction observed in diabetes by studying the critical role of AGE-modified laminin. In future, pharmacological strategies targeted at correcting matrix mobilized AGEs would be helpful to correct Müller cell dysfunction observed in DR.

## Supporting information

S1 Fig GS-1 staining of rMC-1 cells. A representative western blot image showing a prominent band of glutamine synthase 1 (GS-1) in rMC-1 cells.
(TIF)

S2 Fig Cell viability on AGE-modified laminin. The Alamar blue was added after 24 hrs to rMC-1 cells plated on AGE-modified laminin. Bar chart showing AGE-modification of laminin did not affect viability of rMC-1 cells when compared to control, n = 3.
(TIF)

S3 Fig TWIK expression on AGE-modified laminin. Western blot showing a decrease in the levels of TWIK in rMC-1cells treated on AGE-modified laminin.
(TIF)
