# Supplementary figures and images for: Advanced glycation end (AGE) product modification of laminin downregulates Kir4.1 in retinal Müller cells
Source: PLoS One. 2018 Feb 23;13(2):e0193280. doi: 10.1371/journal.pone.0193280 (PMC5825079; doi:10.1371/journal.pone.0193280)

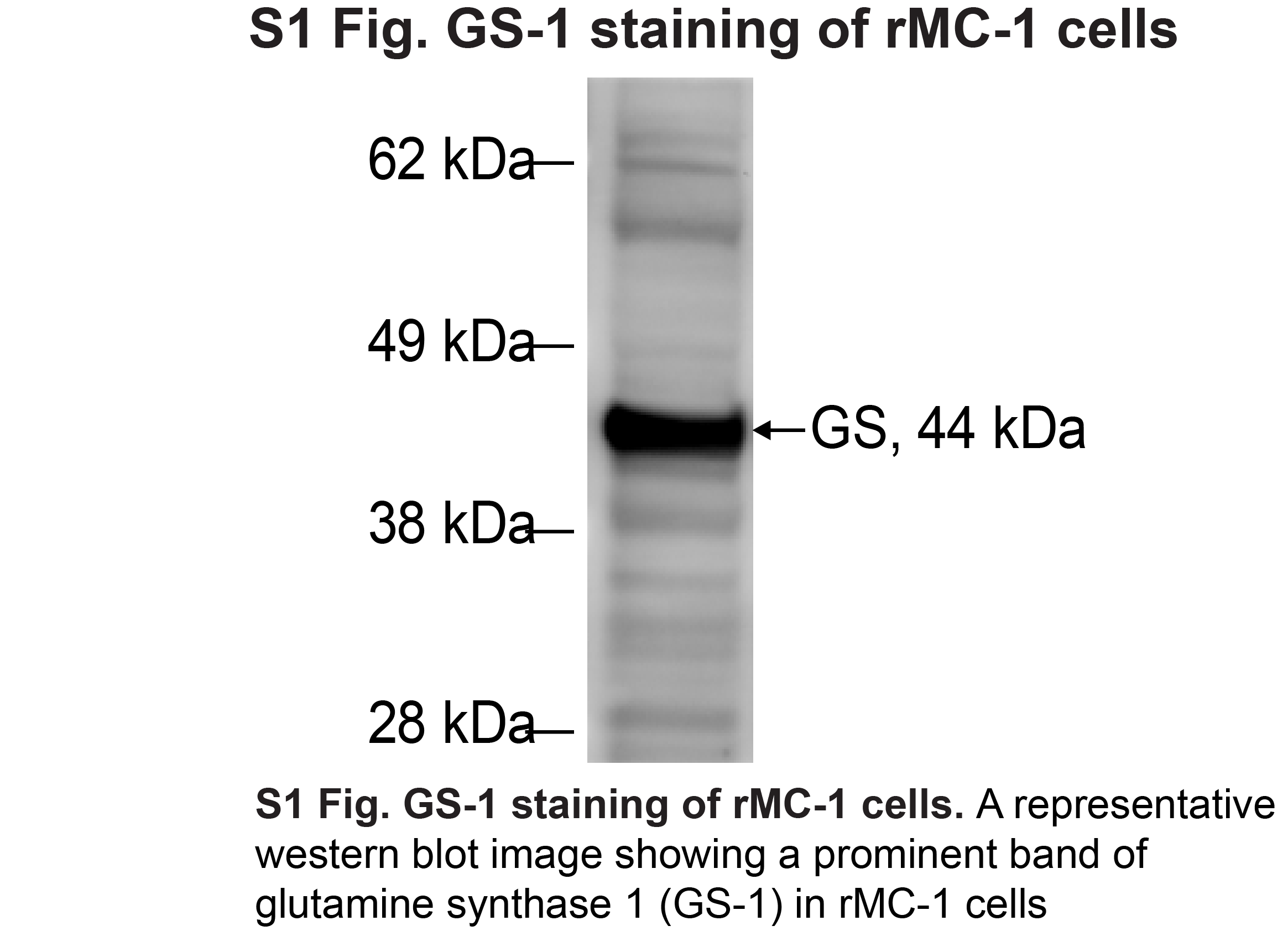

Supplement: S1 Fig — A representative western blot image showing a prominent band of glutamine synthase 1 (GS-1) in rMC-1 cells. (TIF) [file pone.0193280.s001.tif]

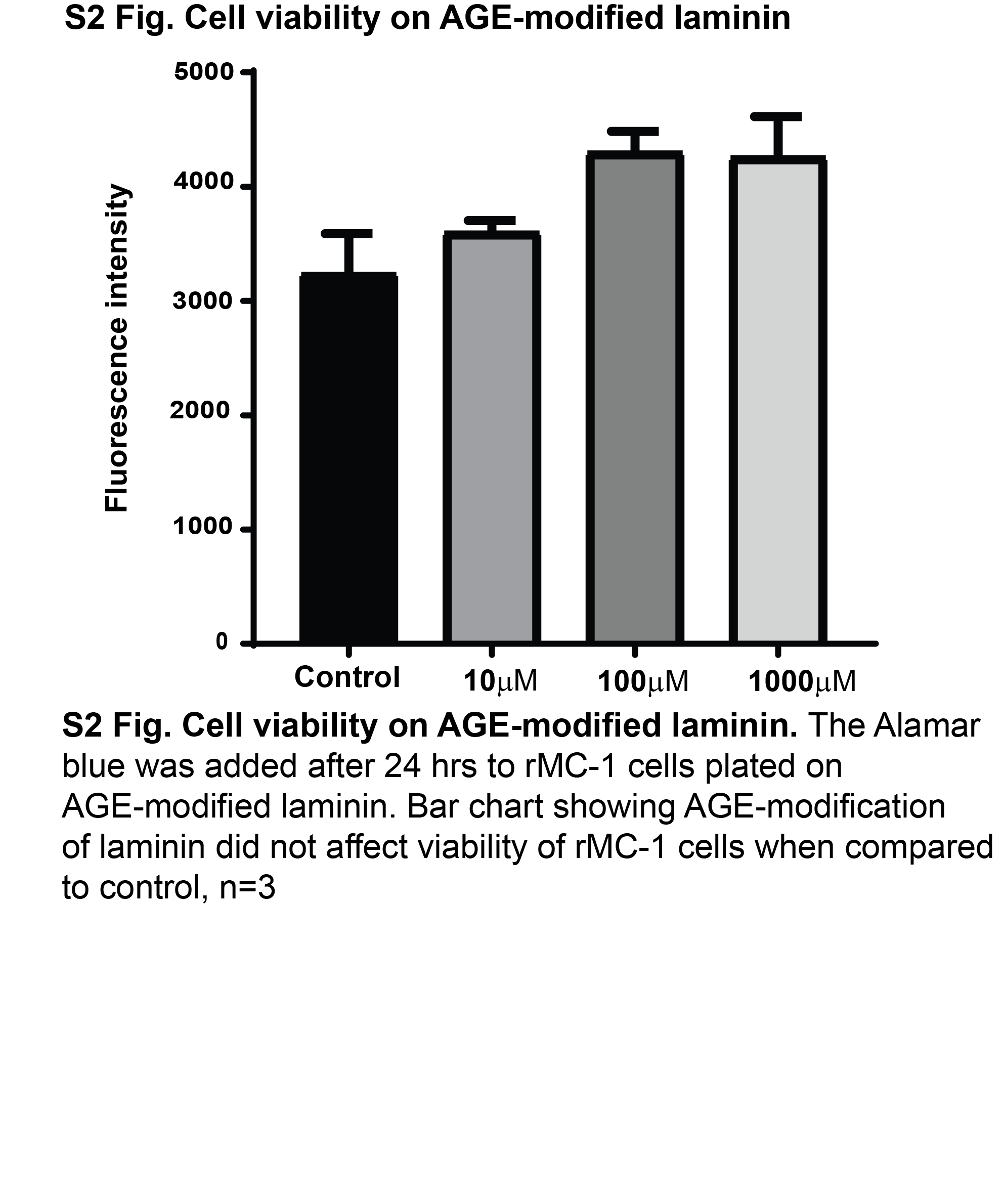

Supplement: S2 Fig — The Alamar blue was added after 24 hrs to rMC-1 cells plated on AGE-modified laminin. Bar chart showing AGE-modification of laminin did not affect viability of rMC-1 cells when compared to control, n = 3. (TIF) [file pone.0193280.s002.tif]

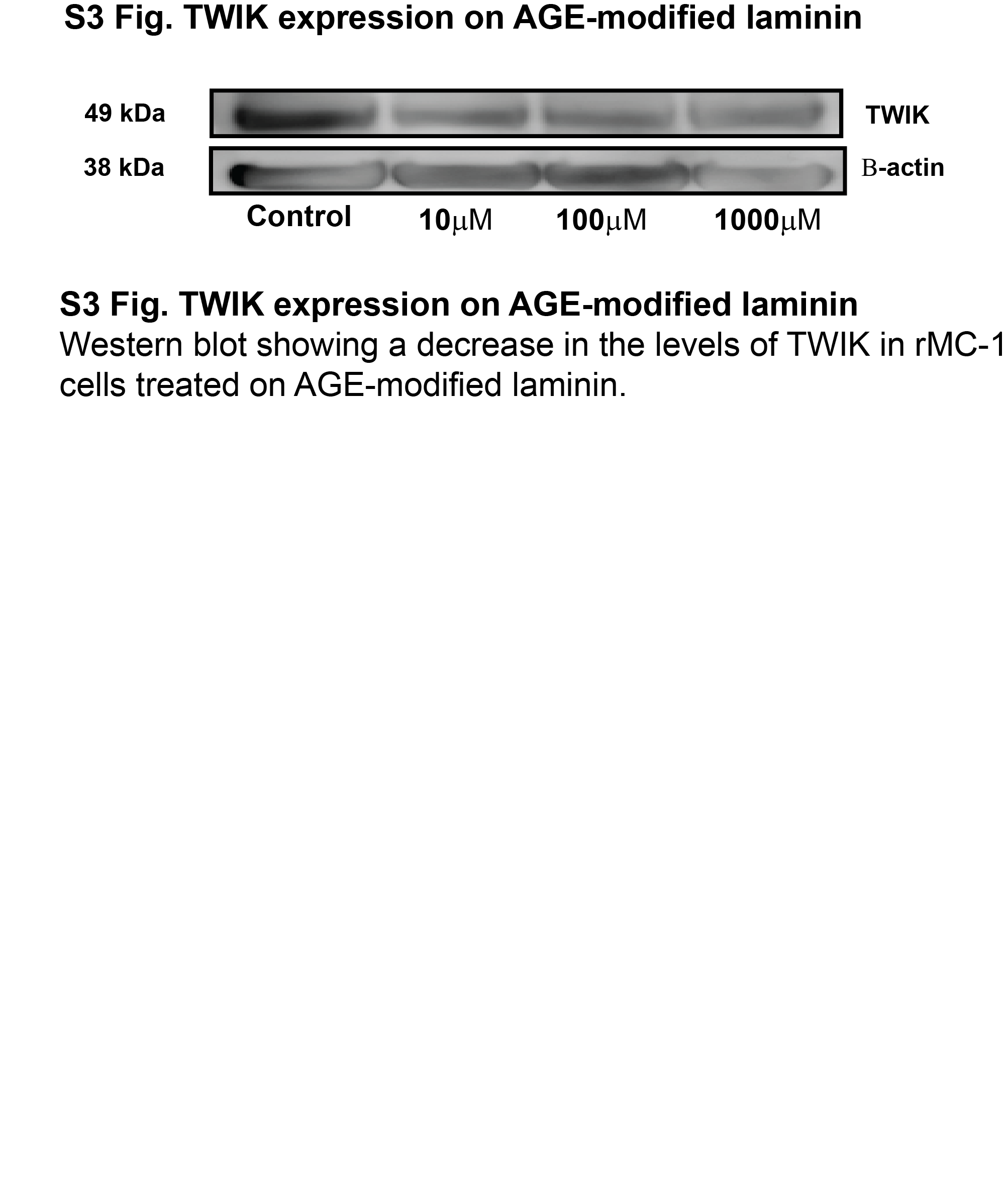

Supplement: S3 Fig — Western blot showing a decrease in the levels of TWIK in rMC-1cells treated on AGE-modified laminin. (TIF) [file pone.0193280.s003.tif]
